# Conserved function of bat IRF7 in activating antiviral innate immunity: insights into the innate immune response in bats

**DOI:** 10.1186/s13567-025-01490-3

**Published:** 2025-03-19

**Authors:** Jie Wang, Qiuju Liu, Caixia Xu, Feiyu Fu, Qi Shao, Yapeng Fu, Zhaofei Wang, Jingjiao Ma, Hengan Wang, Yaxian Yan, Jianhe Sun, Yuqiang Cheng

**Affiliations:** Shanghai Key Laboratory of Veterinary Biotechnology, School of Agriculture and Biology, Shanghai Jiao Tong University, Shanghai, China

**Keywords:** Bat, IRF7, virus, innate immunity, IFN-β

## Abstract

Bats are natural hosts for various highly pathogenic viruses, which pose a considerable threat to humans and animals. However, they rarely display signs of disease infection from these viruses. The expression of IRF7-induced IFN-β plays a crucial role in preventing viral infections. However, the role of bat IRF7 during viral infection remains unclear. In this study, we cloned *Tadarida brasiliensis* IRF7 and discovered that its amino acid sequence was poorly conserved among species. Next, we investigated the expression of bat IRF7 mRNA in *Tadarida brasiliensis* lung cells (TB 1 Lu) infected with RNA viruses such as Newcastle disease virus (NDV), avian influenza virus (AIV), vesicular stomatitis virus (VSV), and the double-stranded RNA (dsRNA) analogue poly (I:C) and demonstrated that these viral infections significantly upregulated the mRNA expression of bat IRF7. Furthermore, the overexpression of IRF7 in TB1 Lu cells activated the expression of bat innate immune-related genes and inhibited virus replication. Importantly, we observed that bat IRF7 function is highly conserved in avian and mammalian species. Structurally, we revealed that the IRF domain of bat IRF7 is essential for activating IFN-β. In summary, our findings indicate that bat IRF7 has a conserved ability to activate bat antiviral innate immunity. This study provides a theoretical foundation for further understanding the innate immune response in bats.

**Supplementary Information:**

The online version contains supplementary material available at 10.1186/s13567-025-01490-3.

## Introduction

Bats, which belong to the order Chiroptera, are a diverse group of mammals comprising a significant portion of the total mammal species [1]. With over 1400 known species, bats are unique not only in that they are the only mammals capable of sustained flight but also because they are natural reservoirs of viruses [2]. Bats harbour several deadly viruses, such as henipaviruses (Hendra and Nipah), rabies, ebola virus, and coronaviruses (54% of those known to be associated with bats): severe acute respiratory syndrome (SARS) coronavirus, Middle East respiratory syndrome (MERS) coronavirus, and the recently emerged severe acute respiratory syndrome coronavirus 2 (SARS-CoV-2) [3–6]. Despite being carriers of these deadly viruses, bats rarely exhibit symptoms of the disease [7]. These findings suggest that bats possess a unique immune system that allows them to coexist with these pathogens. Therefore, the antiviral innate immune regulatory mechanism of bats has attracted increasing attention.

Upon viral infection, pattern recognition receptors (PRRs) recognize the pathogen-associated molecular patterns (PAMPs) of the invading virus and trigger a series of signalling cascade reactions that activate the expression of interferons (IFNs) to control viral replication [8]. IFN expression is regulated by interferon regulatory factors (IRFs). IRFs are a large family of transcription factors that consists of nine members [9]. They, through a helix-turn-helix DNA-binding motif, bind to the IFN-stimulated consensus response element (ISRE) in the promoter region of IFN genes to regulate IFN transcription [10]. Among them, IRF1, IRF3, IRF5, and IRF7 have been identified as positive regulators of type I IFN transcription [11, 12]. IRF7 is considered a master regulator of type I IFN production [13]. It was initially cloned within the biological context of Epstein–Barr virus (EBV) latency and was discovered to have an intimate relationship with the EBV primary oncogenic protein latent membrane protein-1 (LMP-1) [14]. Studies using mice deficient in the IRF7 gene have shown that IRF7 is essential for the induction of IFN-alpha/beta genes, while IRF3 also plays a role in these pathways, and its contribution is minimal in the absence of IRF7 [13]. In humans, IRF7 predominantly acts on plasmacytoid dendritic cells (pDCs) through the activation of TLR7/9 and the MyD88-dependent signalling pathway [15]. In chickens, where IRF3 is absent, IRF7 is utilized to reconstitute the corresponding IFN signalling pathway in response to viral infection [16]. These findings underscore the importance of IRF7 in innate immunity and its crucial role in regulating the production of type I IFNs.

Studies have shown that bat IRF1/3/7 have high basal expression [17], which means that bats can rapidly and effectively initiate an innate immune response upon viral infection. Our previous studies revealed that bat IRF1 can activate IFN-β expression and inhibit viral replication [18]. Another study revealed that IRF3-mediated signalling limits MERS coronavirus propagation in cells from an insectivorous Bat [19]. This mechanism helps prevent excessive replication of MERS coronavirus, which is crucial for host health and contributes to the ability of bats to coexist with these viruses. However, researchers have not clearly determined whether bat IRF7 also plays a conserved role in antiviral innate immunity.

In this study, we cloned the IRF7 gene from *Tadarida brasiliensis* cells and discovered that the amino acid sequence of bat IRF7 was poorly conserved among species. However, we observed that RNA viruses were able to significantly increase the expression of IRF7 mRNA in TB 1 Lu cells. Furthermore, the overexpression of bat IRF7 not only activated the bat IFN-β promoter but also activated the IFN-β promoters of chickens and humans. Additionally, the overexpression of bat IRF7 was found to increase the expression of genes related to innate immunity in TB 1 Lu cells and inhibit virus replication. Overall, these findings suggest that bat IRF7 plays a conserved role in antiviral innate immunity. These findings enhance our understanding of innate immunity in bats and shed light on the mechanisms underlying the coexistence of bats and viruses.

## Materials and methods

### Cell culture and virus

The chicken embryonic fibroblast line DF1, human 293T cells, and bat TB1 Lu cells were obtained from ATCC and cultured in DMEM supplemented with 10% FBS, after which the cells were incubated at 37 °C in a 5% CO_2_ incubator. Newcastle disease virus (NDV-GFP) is a low-virulence strain of LaSota named NDV-GFP. The avian influenza virus (AIV) used was A/Chicken/Shanghai/010/2008 (H9N2) virus (SH010), which was isolated from chickens in Shanghai, China, in 2008 and identified as H9N2 avian influenza A virus. The GFP-tagged vesicular stomatitis virus (VSV) VSV-GFP was stored in our laboratory. The viruses were purified, propagated, and stored as described in our previous study [20].

### Virus infection and poly(I:C) stimulation

TB 1 Lu cells were seeded into 12-well culture plates at a density of 2.5 × 10^4^, and when the cultures reached 80% confluence, the culture medium was replaced with fresh serum-free DMEM and infected with NDV-GFP or AIV or VSV-GFP at an MOI of 1.0 or transfected with poly (I:C) at 0.1 μg/mL. After 3 h, 12 h, and 24 h of infection, samples were collected for subsequent experiments.

### Cloning and bioinformatics analysis of batIRF7

On the basis of the Molossus molossus IRF7 sequence (XM_036281692.1) obtained from the National Center for Biotechnology Information (NCBI), the primers bat IRF7-F and bat IRF7-R (Additional file 1) were designed and used to amplify bat IRF7 from TB 1 Lu cell cDNA. The PCR product was ligated into a pTOPO-Blunt vector (Vazyme Biotech Co., Ltd.) for sequencing, and the positive colonies were sent to the Beijing Genomics Institute (Beijing, China) for sequencing. The amino acid sequence of bat IRF7 was aligned with those of other animal IRF7 proteins from chickens, ducks, pigs, cattle, dogs, cats, mice, humans, zebrafish, and salmon via ClustalW and edited with ESPript 3.0. Sequence homology and phylogenetic analysis of the IRF7 amino acid sequences were conducted using DNASTAR. A phylogenetic tree was constructed on the basis of IRF7 from 15 different species, including mammals, birds, and fish. Different domains in the IRF7 amino acid sequence were predicted using the simple modular architecture research tool (SMART) program. Homology modelling for IRF7 was conducted via the online protein-modelling server Swiss Model.

### Plasmid construction

pcDNA3.1-bat-IRF7 plasmids were constructed by inserting full-length *Tadarida brasiliensis* IRF7 into the *Xho*I and *EcoR*I sites of the expression vector pcDNA3.1 via a ClonExpress II one-step cloning kit (Yeasen, Shanghai, China). The primers used in the PCR are listed in Additional file 1. The truncated plasmids of bat IRF7, including those in which amino acids 123–223 (dAA123-223), 223–335 (dAA223-335), insulin growth factor-binding protein homologues (IB), interferon regulatory factor (IRF), interferon-regulatory factor 3 (IRF-3), and repeats of unknown function (DUF) were deleted separately, were constructed using a modified homologous recombination method, and the primers used are listed in Additional file 1. DH5α chemically competent cells (Tsingke Biology Technology, Beijing, China) were used for plasmid transformation. The pGL-IFN-β-Luc plasmid was constructed in our previous study [21].

### Cell transfection

293T, DF1 and TB 1 Lu cells were seeded in 12-well or 24-well plates (NEST Biotechnology, Wuxi, China) at 5 × 10^5^/mL or 1 × 10^6^/mL. The plasmid was transfected at 250 ng/well in 24-well plates or 500 ng/well in 12-well plates. Plasmid transfection was performed with Nulen Plus-Trans™ Transfection Reagent (Nulen, Shanghai, China) according to the manufacturer’s protocol.

### Luciferase reporter assay

DF-1, 293T, and TB 1 Lu cells were plated in 24-well plates, and after reaching 80% confluence, the cells were transiently co-transfected with 1) the target plasmid pcDNA3.1-bat-IRF7 plasmids or truncated plasmids of bat IRF7 (250 ng/well), 2) the reporter plasmid pGL-chIFN-β-Luc or pGL-huIFN-β-Luc or pGL-batIFN-β-Luc (120 ng/well) or 3) the control Renilla luciferase (pRL-TK, 60 ng/well). The selection of the reporter plasmid PGL-IFN-β-Luc should correspond to the cell. The cells were lysed 24 h after transfection, and luciferase activity was detected via a Dual-Luciferase Reporter Assay System Kit (Promega, Madison, WI, USA) according to the manufacturer’s instructions. Renilla luciferase activity was used for normalization.

### RNA extraction and quantitative real-time PCR

Total RNA was extracted from the cells with AG RNAex Pro Reagent (Ac-curate Biology, Hunan, China). The mRNA was reverse transcribed to cDNA via a two-step reverse transcription kit. Genomic DNA was removed from the first reaction via the addition of gDNA wiper enzyme, and the second step involved the reverse transcription of the mRNA to cDNA. The specific operation was carried out according to the instructions provided by the kit (Vazyme, Nanjing, China), and the cDNA was analysed via SYBR Green PCR mix (Vazyme) with an Applied Biosystems instrument (ABI 7500; Thermo Fisher Scientific). Relative gene expression was analysed via the 2^−ΔΔCt^ method. β-actin was used as the internal reference when the levels of genes were examined. The primer sequences for the genes are shown in Additional file 1.

### Western blot analysis

The total protein was extracted from the cells via a radioimmunoprecipitation assay (Beyotime, Shanghai, China) containing a protease cocktail (Yeasen) and phenylmethylsulfonyl fluoride (PMSF) (Yeasen). The lysate was centrifuged at 13 000 rpm for 10 min to obtain the supernatant, and 5 × SDS loading buffer was added before the lysates were boiled for 10 min. The proteins isolated from the cell lysates were separated via SDS‒PAGE and analysed using Western blotting. The antibodies used included anti-GFP (Yeasen) and β-tubulin, which were incubated overnight at 4 °C. The membrane was washed 3 times with Tris-buffered saline and Tween-20 (TBST) (Sangon Biotech Co., Ltd., Shanghai, China). Then, the secondary antibody was added, and the samples were incubated for 1 h at 4 °C on a shaker. After being washed three times with TBST, the membrane was placed into the developer for approximately 1 min. Images were obtained using a Tanon 5200 imaging system (Tanon, Shanghai, China).

### Statistical analysis

The results are expressed as the means ± SD. GraphPad Prism 8.0 was used to generate graphs of the results. The data were analysed via a two-tailed independent Student’s *t* test. *P* < 0.05 was considered statistically significant, and *P* < 0.01 was considered highly statistically significant (*P < 0.05; **P < 0.01).

## Results

### Upregulation of bat IRF7 expression in response to RNA viral infection

**Figure 1:**
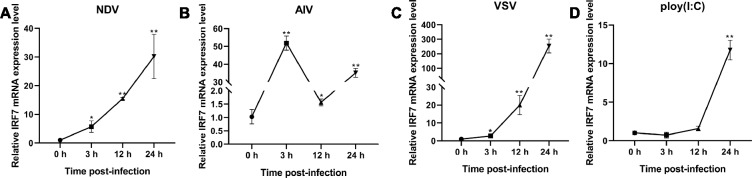
**Upregulation of bat IRF7 expression in response to RNA viral infection.** **A** RT‒qPCR was used to detect the mRNA expression level of bat IRF7 in TB 1 Lu cells infected with NDV at an MOI of 1.0 for 0, 3, 12, or 24 h. **B** RT‒qPCR was used to detect the mRNA expression level of bat IRF7 in TB 1 Lu cells infected with AIV at an MOI of 1.0 for 0, 3, 12, or 24 h. **C** RT‒qPCR was used to detect the mRNA expression level of bat IRF7 in TB 1 Lu cells infected with VSV at an MOI of 1.0 for 0, 3, 12, or 24 h. **D** RT‒qPCR was used to detect the mRNA expression level of bat IRF7 in TB 1 Lu cells stimulated with 0.1 μg/mL poly(I:C) for 0, 3, 12, or 24 h. Data are expressed as the mean ± SD of three independent experiments. *P < 0.05; **P < 0.01.

Mammalian and avian cells infected with RNA viruses exhibit significant up-regulation of IRF7 expression, which plays a crucial role in controlling virus replication [22, 23]. However, it remains unclear whether RNA virus infection can also induce the upregulation of IRF7 in bats. To investigate the role of bat IRF7 in RNA virus infection, we infected bat TB 1 Lu cells with Newcastle disease virus (NDV), avian influenza virus (AIV), and vesicular stomatitis virus (VSV-GFP). The expression of bat IRF7 mRNA was assessed at 0 h, 3 h, 12 h, and 24 h post-infection. Our findings revealed that all three RNA viruses significantly upregulated the expression of bat IRF7 after 3 h of infection (Figures 1A–C). To further confirm the universality of this effect, we transfected TB 1 Lu cells with an RNA virus nucleic acid mimic, poly(I:C), and observed that it also induced the expression of IRF7 in bats (Figure 1D). These results provide evidence that virus infection of bat cells can indeed upregulate the expression of IRF7. These findings suggest that IRF7 has the potential to participate in anti-RNA virus immune responses.

### Bioinformatic analysis of bat IRF7

**Figure 2:**
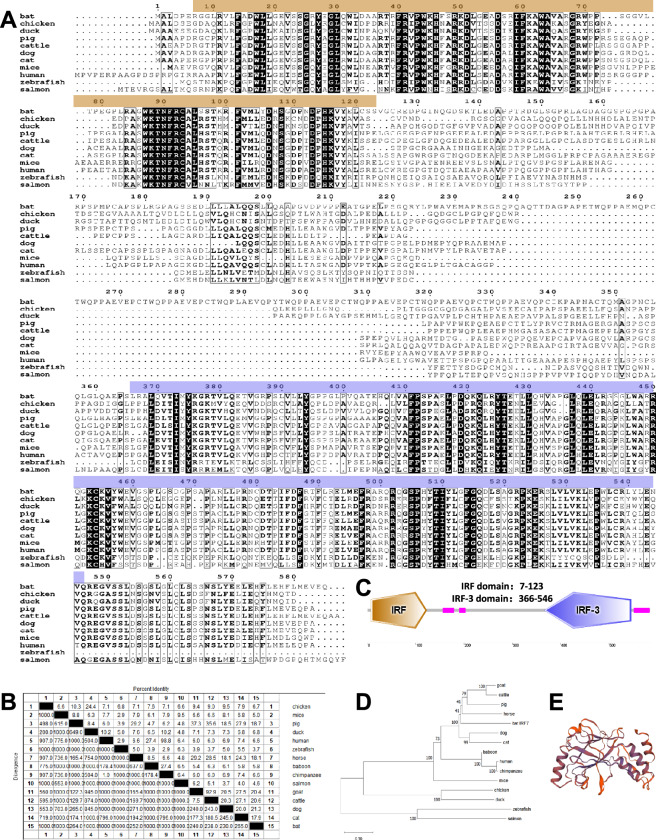
**Bioinformatic analysis of bat IRF7.** **A** Alignment of IRF7 amino acid sequences in different species, including bats (*Tadarida brasiliensis*), chickens (AJS11515.1), ducks (AYI50403.1), pigs (NP_001090897.1), cattle (AAI51519.1), dogs (XP_038279968.1), cats (XP_011285476.2), mice (NP_058546.1), humans (AAI36556.1), zebrafish (AAH65902.1) and salmon (NP_001165321.1). The amino acid sequences of different animals were aligned using ClustalW and edited with ESPript 3.0. **B** Homology analysis of IRF7 amino acid sequences in different species. **C** Protein domains of bat IRF7 predicted by SMART. **D** Phylogenetic tree of vertebrate IRF7. A neighbor-joining phylogenetic tree of vertebrate IRF7 was generated with MegAlign software using IRF7 sequences from the following animals: chickens (AJS11515.1), mice (NP_058546.1), pigs (NP_001090897.1), ducks (AYI50403.1), humans (AAI36556.1), zebrafish (AAH65902.1), horses (XP_023510519.1), baboons (XP_031509712.1), chimpanzees (JAA38191.1), salmon (NP_001165321.1), goats (XP_017898523.1), cattle (AAI51519.1), dogs (XP_038279968.1), cats (XP_011285476.2) and bats (*Tadarida brasiliensis*). **E** Three dimensional structure of bat IRF7 predicted using SWISS-MODEL.

To elucidate the biological function of bat IRF7 in antiviral innate immunity, we cloned the *Tadarida brasiliensis* bat IRF7 from the *Tadarida brasiliensis* 1 lung (TB 1 Lu) cell line cDNA. The open reading frame (ORF) of IRF7 was found to be 1758 bp long, encoding 586 amino acids. Our multiple sequence alignment analysis revealed that bat IRF7 is poorly conserved among different species. The similarity of bat IRF7 with various species, including chickens (AJS11515.1), mice (NP_058546.1), pigs (NP_001090897.1), ducks (AYI50403.1), humans (AAI36556.1), zebrafishes (AAH65902.1), horses (XP_023510519.1), baboons (XP_031509712.1), chimpanzees (JAA38191.1), salmon (NP_001165321.1), goats (XP_017898523.1), cattle (AAI51519.1), dogs (XP_038279968.1) and cats (XP_011285476.2), ranged from 3.7 to 21.3% (Figures 2A and B). Furthermore, we performed structural analysis and identified four characteristic domains in bat IRF7: a factor-binding protein homologue (IB) domain, an interferon regulatory factor (IRF) domain, an interferon-regulatory factor 3 (IRF-3) domain, and a repeat of unknown function (DUF) domain (Figure 2C). To explore the evolutionary relationships of bat IRF7 with those of other species, we conducted a phylogenetic analysis based on multiple alignments of IRF7 from mammals, birds, and fishes. Our analysis revealed that bat IRF7 belongs to a subgroup with other mammals, whereas birds and fishes have distinct subgroups (Figure 2D). Additionally, we used the Swiss model to predict the three-dimensional structure of bat IRF7, which revealed the presence of 4 α-helices and 11 β-folds (Figure 2E). Overall, our study provides a comprehensive characterization and evolutionary analysis of bat IRF7, shedding light on its potential regulatory effects on antiviral innate immunity.

### Overexpression of bat IRF7 activates the innate immune response of bats

**Figure 3:**
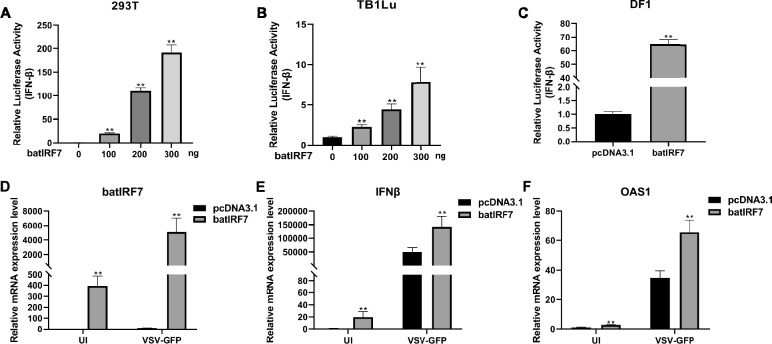
**Overexpression of bat IRF7 significantly activated antiviral innate immunity.** **A** 293T cells were transiently transfected with the IFN-β reporter plasmid with increasing amounts of bat IRF7 plasmid (0 ng/well, 100 ng/well, 200 ng/well, or 300 ng/well), and luciferase activity was analysed. **B** TB 1Lu cells were transiently transfected with the IFN-β reporter plasmid with increasing amounts of bat IRF7 plasmid (0 ng/well, 100 ng/well, 200 ng/well, or 300 ng/well), and their luciferase activity was analysed. **C** DF1 cells were transiently transfected with the IFN-β reporter plasmid and the bat IRF7 plasmid, and luciferase activity was analysed. **D** The efficiency of bat IRF7 overexpression in TB 1 Lu cells was assessed by RT‒qPCR. The cells were transfected with 500 ng/well of pcDNA3.1 or pcDNA3.1-bat-IRF7 and then uninfected (UI) or infected with VSV-GFP at an MOI of 1.0. **E**, **F** The expression levels of IFN-β and OAS1 were detected by RT‒qPCR in TB 1 Lu cells after the overexpression of pcDNA3.1-bat IRF7. The cells were uninfected (UI) or infected with VSV-GFP at an MOI of 1.0. The data are presented as the mean ± SD of three independent experiments. *P < 0.05; **P < 0.01.

To further explore the role of bat IRF7 in antiviral innate immunity, pcDNA3.1-bat-IRF7 plasmids and luciferase reporter plasmids (pRL-TK and pGL-IFN-β-Luc) were cotransfected into TB 1 Lu and 293T cells. Dual-luciferase reporter assays were subsequently performed to assess the activity of the IFN-β promoter. The results revealed that the overexpression of bat IRF7 significantly activated IFN-β promoter activity in a dose-dependent manner in both 293 T and TB 1 Lu cells (Figures 3A and B). Additionally, we overexpressed bat IRF7 in DF1 cells and found that bat IRF7 can also significantly activate the chicken IFN-β promoter (Figure 3C), indicating that bat IRF7 has a conserved role in activating IFN-β in both mammals and birds.

Furthermore, we examined the effect of bat IRF7 on the innate immune response of bats. When bat IRF7 was overexpressed in bat TB 1 Lu cells, we observed significant increases in the expression of IFN-β and OAS1 both before and after VSV-GFP virus infection (Figures 3D–F). These findings suggest that bat IRF7 can activate the innate immune response in bats.

### Bat IRF7 inhibits vesicular stomatitis virus (VSV-GFP) replication

**Figure 4:**
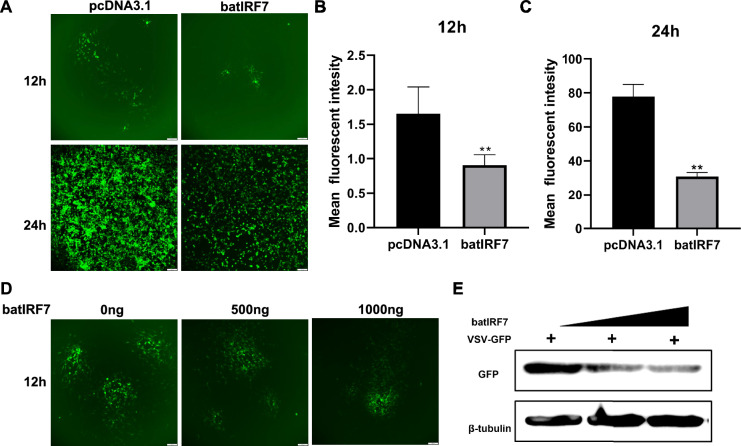
**Overexpression of bat IRF7 inhibits viral replication.** **A** Viral fluorescence in TB 1 Lu cells after overexpression of pcDNA3.1 or pcDNA3.1-bat-IRF7 and infection with VSV-GFP at an MOI of 1.0 for 12 and 24 h. **B**, **C** Mean fluorescence intensity of VSV-GFP in TB 1 Lu cells after overexpression of pcDNA3.1 or pcDNA3.1-bat IRF7 and infection with VSV-GFP at an MOI of 1.0 for 12 h (**B**) or 24 h (**C**). **D** Viral fluorescence in TB 1 Lu cells after the overexpression of bat IRF7 at 0 ng/well, 500 ng/well, or 1000 ng/well and infection with VSV-GFP at an MOI of 1.0 for 12 h. **E** Western blot analysis was used to detect the expression of GFP, which reflects virus replication, after the overexpression of bat IRF7 at 0 ng/well, 500 ng/well, or 1000 ng/well and infection with VSV-GFP at an MOI of 1.0 for 12 h. Data are expressed as the mean ± SD of three independent experiments. *P < 0.05; **P < 0.01.

To investigate the impact of bat IRF7 on virus replication, we overexpressed bat IRF7 in bat TB 1 Lu cells and infected TB 1 Lu with vesicular stomatitis virus (VSV-GFP). The replication of the virus was observed at 12 and 24 h post-infection. The expression of GFP reflects the amount of virus replication. Fluorescence microscopy analysis revealed that the overexpression of bat IRF7 reduced the fluorescence intensity of the virus (Figure 4A). Furthermore, quantification of the fluorescence intensity confirmed that the overexpression of bat IRF7 significantly inhibited the replication of the VSV-GFP virus (Figure 4B). Additionally, as the concentration of bat IRF7 increased, the fluorescence intensity of VSV-GFP gradually decreased (Figure 4C). Western blot analysis further demonstrated a concentration-dependent decrease in the GFP level with increasing bat IRF7 concentration (Figure 4D). These results suggest that bat IRF7 can activate the innate immune response in bats to inhibit virus replication.

### Essential domains of bat IRF7 in IFN-β activation

**Figure 5:**
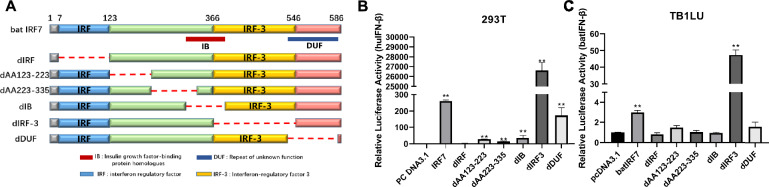
**Essential functional domains of bat IRF7**. **A** Schematic structure of bat IRF7 mutants lacking the AA123-223, AA223-335, IB, IRF, IRF3, or DUF domains. **B** A luciferase reporter assay was performed to measure IFN-β promoter activity in 293T cells. The cells were co-transfected with luciferase reporter plasmids (pRL-TK and pGL-huIFN-β-Luc) along with bat IRF7 mutant plasmid or pcDNA3.1 control. After 6 h of transfection, the cells were infected with VSV-GFP at an MOI of 1.0 for 16 h. A dual-luciferase reporter assay was used to detect IFN-β promoter activity. **C** A luciferase reporter assay was performed to measure the IFN-β promoter activity in TB 1 Lu cells. The cells were co-transfected with luciferase reporter plasmids (pRL-TK and pGL-batIFN-β-Luc) along with bat IRF7 mutant plasmid or pcDNA3.1 control. After 6 h of transfection, the cells were infected with VSV-GFP at an MOI of 1.0 for 16 h. A dual-luciferase reporter assay was used to detect IFN-β promoter activity. The data are presented as the mean ± SD of three independent experiments. *P < 0.05; **P < 0.01.

These studies indicate that bat IRF7 has a conserved ability to activate innate immunity and inhibit virus replication. However, the structural basis by which IRF7 functions is unclear. To further analyse its important functional domains, we constructed a series of plasmids with deletions of different functional domains, including the IRF7 AA123-223, AA223-335, IB, IRF, IRF3, and DUF domains (Figure 5A). These plasmids were transfected into 293T and TB 1 Lu cells, and the deletion of the IRF7 AA123-223, AA223-335, IB, and DUF domains weakened the ability of IRF7 to activate the IFN-β promoter. Conversely, the deletion of the IRF3 domain significantly enhanced its activation of IFN-β promoter activity in both 293T and TB 1 Lu cells (Figures 5B and C). These findings suggest that the IRF domain, AA123-223, AA223-335, and IB domain are essential for bat IRF7 to activate IFN-β, whereas the IRF3 domain acts as an inhibitor.

## Discussion

As natural reservoirs of numerous highly pathogenic viruses, bats significantly threaten human health and survival. However, what is intriguing is that bats themselves do not exhibit any apparent signs of disease despite carrying such viruses [24]. This phenomenon suggests that bats possess specific and unique innate immune characteristics. Multiple studies have revealed that bats have greater basal expression of interferons (IFNs) and interferon-stimulated genes (ISGs) than other animals do. This heightened immune response may play a crucial role in bats’ ability to respond effectively to viral infections and maintain a virus tolerance phenotype [25]. However, the precise regulatory mechanisms underlying this high basal expression and its potential effects on bats’ immune response require further investigation.

Members of the IRF family play important roles in regulating the expression of IFNs and ISGs. In particular, IRF3 and IRF7 can significantly induce IFN-α/β gene transcription [26]. In mammals, IRF3 is a constitutive gene that is expressed in various tissues and organs, whereas IRF7 is an inducible gene that is strongly induced by IFN [9]. In birds, IRF3 is naturally missing, and IRF7 plays a major role [16, 23]. However, both IRF3 and IRF7 are constitutive genes in bats, and they have relatively high basal expression. These findings suggest that bat IRF7 has unique functions that remain to be defined.

In this study, we cloned *Tadarida brasiliensis* IRF7. Analysis of the bat IRF7 amino acid sequence revealed that the middle region sequence of IRF7 has extremely low conservation. However, the N-terminal DNA binding domain (DBD) and C-terminal sequence are relatively conserved. The DBD at the N-terminus is particularly important for the function of IRF as a transcription factor [27]. The DBD contains a unique cluster of five well-spaced tryptophan residues, and fish possess only four of the five conserved tryptophan residues [28, 29]. This region forms a helix-turn-helix motif that latches onto DNA-recognizing elements containing GAAA repeats [30]. These five tryptophan sites in bat IRF7 are conserved. The C-terminal region of mammalian IRF7 contains a conserved serine-rich domain important for virus-induced phosphorylation. Both human and mouse IRF7 have two serine sites that are phosphorylated in this region [31]. The serine-rich domain is also conserved in bats. These findings indicate that the key functional domains of bat IRF7 are highly conserved among species. Bats have evolved unique immune systems over tens of thousands of years, and bats’ immune systems exhibit some of the conserved characteristics common to those of other mammals [32]. It exhibits unique features that have evolved to adapt to virus infections, such as lower NLRP3 production, greater expression of IFNα1, and greater phosphorylation of IRF3 [33]. These findings indicate that the function of bat IRF7 may also be conserved during species evolution.

To explore the function of bat IRF7, we detected the basal expression of batIR7 and its induced expression after virus infection. Three RNA viruses, Newcastle disease virus (NDV), avian influenza virus (AIV), and vesicular stomatitis virus (VSV), significantly upregulated bat IRF7 mRNA expression after infection of TB 1 Lu cells. In addition, the overexpression of bat IRF7 significantly upregulated the expression of bat innate immunity-related genes and inhibited virus replication. Similar results were found in Australian black flies: when bat IRF7 was knocked down, the Pulau virus replicated to a titre more than fourfold greater than that in mock-transfected cells. These findings suggest that bat IRF7, like other animal IRF7s, also has typical features of interferon stimulation-related genes (ISGs). In this study, we also revealed that bat IRF7 not only activates the bat IFN-β promoter but also activates the chicken and human IFN-β promoters. These findings suggest that bat IRF7 has conserved functions with other animal IRF7s. Studies have shown that Bat IRF7 is expressed constitutively and has relatively high basal expression. Higher basal expression facilitates a stronger antiviral response. This may help explain why bats coexist with a variety of viruses [17]. Humans and bats vary in the type of response and the symptoms caused by infections. Al‐Eitan et al. suggested that bat IFNs could be used to enhance antiviral responses in other species, especially humans [33]. Bat IRF7, as a regulator of IFNs, can significantly activate human IFN-β expression. Its potential in drug development deserves attention.

Structurally, IRF7 contains a C-terminal IRF3 domain (AA366–546), which may explain why IRF7 has greater homology with IRF3 than with other IRFs. After the deletion of the IRF3 domain, the ability of IRF7 to activate IFN-β was greatly improved. These findings indicate that the IRF3 domain is inhibitory. In humans, the C-terminal region of IRF-7 also contains an inhibitory domain (ID) that interferes with its transactivation function. This finding is consistent with what we mentioned earlier: the C-terminus of IRF7 is relatively conserved among species. When the N-terminal IRF domain was deleted, the activation of IFN-β by bat IRF7 was completely lost. These findings indicate that the N-terminal IRF7 domain is crucial for the function of IRF7 as a transcription factor. The conserved cluster of five well-spaced tryptophan residues associated with DNA binding is located in this domain.

In conclusion, the amino acid sequence of bat IRF7 is relatively poorly conserved among species and retains only the key conserved functional domain that activates IFN-β expression. Mutation of the remaining functional domain amino acids may increase the function of bat IRF7 and promote its coexistence with viruses. However, the specific mechanism still needs further study.

Overall, in this study, we found that bat IRF7 has a conserved antiviral innate immune function. This study helps us further understand the innate immune system of bats and provides a target for the development of antiviral drugs based on innate immunity strategies.

## Supplementary Information

**Additional file 1. Sequences of Primers used in this study, including bat IRF7 cloning primers, bat IRF7 truncated plasmid primers, and quantitative RT‒PCR primers.**

## Data Availability

The data analysed during the current study are available from the corresponding author upon reasonable request.

## References

[1] Szentivanyi T, Christe P, Glaizot O (2019) Bat flies and their microparasites: current knowledge and distribution. Front Vet Sci 6:115 31106212 10.3389/fvets.2019.00115 PMC6492627

[2] Hedenstrom A, Johansson LC (2015) Bat flight: aerodynamics, kinematics and flight morphology. J Exp Biol 218:653–663 25740899 10.1242/jeb.031203

[3] Woo PC, Lau SK, Huang Y, Yuen KY (2009) Coronavirus diversity, phylogeny and interspecies jumping. Exp Biol Med (Maywood) 234:1117–1127 19546349 10.3181/0903-MR-94

[4] Bermingham A, Chand MA, Brown CS, Aarons E, Tong C, Langrish C, Hoschler K, Brown K, Galiano M, Myers R, Pebody RG, Green HK, Boddington NL, Gopal R, Price N, Newsholme W, Drosten C, Fouchier RA, Zambon M (2012) Severe respiratory illness caused by a novel coronavirus, in a patient transferred to the United Kingdom from the Middle East, September 2012. Euro Surveill 17:20290 23078800

[5] Cui J, Li F, Shi ZL (2019) Origin and evolution of pathogenic coronaviruses. Nat Rev Microbiol 17:181–192 30531947 10.1038/s41579-018-0118-9 PMC7097006

[6] Temmam S, Vongphayloth K, Baquero E, Munier S, Bonomi M, Regnault B, Douangboubpha B, Karami Y, Chretien D, Sanamxay D, Xayaphet V, Paphaphanh P, Lacoste V, Somlor S, Lakeomany K, Phommavanh N, Perot P, Dehan O, Amara F, Donati F, Bigot T, Nilges M, Rey FA, van der Werf S, Brey PT, Eloit M (2022) Bat coronaviruses related to SARS-CoV-2 and infectious for human cells. Nature 604:330–336 35172323 10.1038/s41586-022-04532-4

[7] Ahn M, Chen VC, Rozario P, Ng WL, Kong PS, Sia WR, Kang AEZ, Su Q, Nguyen LH, Zhu F, Chan WOY, Tan CW, Cheong WS, Hey YY, Foo R, Guo F, Lim YT, Li X, Chia WN, Sobota RM, Fu NY, Irving AT, Wang LF (2023) Bat ASC2 suppresses inflammasomes and ameliorates inflammatory diseases. Cell 186:2144–2159.e22 10.1016/j.cell.2023.03.036 37172565

[8] Karki R, Kanneganti TD (2022) Innate immunity, cytokine storm, and inflammatory cell death in COVID-19. J Transl Med 20:542 36419185 10.1186/s12967-022-03767-z PMC9682745

[9] Negishi H, Taniguchi T, Yanai H (2018) The interferon (IFN) class of cytokines and the IFN regulatory factor (IRF) transcription factor family. Cold Spring Harb Perspect Biol 10:a028423 28963109 10.1101/cshperspect.a028423 PMC6211389

[10] Zhou H, Tang YD, Zheng C (2022) Revisiting IRF1-mediated antiviral innate immunity. Cytokine Growth Factor Rev 64:1–6 35090813 10.1016/j.cytogfr.2022.01.004

[11] Honda K, Takaoka A, Taniguchi T (2006) Type I interferon [corrected] gene induction by the interferon regulatory factor family of transcription factors. Immunity 25:349–360 16979567 10.1016/j.immuni.2006.08.009

[12] Paun A, Pitha PM (2007) The IRF family, revisited. Biochimie 89:744–753 17399883 10.1016/j.biochi.2007.01.014 PMC2139905

[13] Honda K, Yanai H, Negishi H, Asagiri M, Sato M, Mizutani T, Shimada N, Ohba Y, Takaoka A, Yoshida N, Taniguchi T (2005) IRF-7 is the master regulator of type-I interferon-dependent immune responses. Nature 434:772–777 15800576 10.1038/nature03464

[14] Zhang L, Pagano JS (2002) Structure and function of IRF-7. J Interferon Cytokine Res 22:95–101 11846980 10.1089/107999002753452700

[15] Ahad A, Smita S, Mishra GP, Biswas VK, Sen K, Gupta B, Garcin D, Acha-Orbea H, Raghav SK (2020) NCoR1 fine-tunes type-I IFN response in cDC1 dendritic cells by directly regulating Myd88-IRF7 axis under TLR9. Eur J Immunol 50:1959–1975 32644192 10.1002/eji.202048566

[16] Cheng Y, Zhu W, Ding C, Niu Q, Wang H, Yan Y, Sun J (2019) IRF7 is involved in both STING and MAVS mediating IFN-beta signaling in IRF3-lacking chickens. J Immunol 203:1930–1942 31366714 10.4049/jimmunol.1900293

[17] Irving AT, Zhang Q, Kong PS, Luko K, Rozario P, Wen M, Zhu F, Zhou P, Ng JHJ, Sobota RM, Wang LF (2020) Interferon regulatory factors IRF1 and IRF7 directly regulate gene expression in bats in response to viral infection. Cell Rep 33:108345 33147460 10.1016/j.celrep.2020.108345 PMC8755441

[18] Liu Q, Zhang M, Wang J, Zhang J, Wang Z, Ma J, Yan Y, Sun J, Cheng Y (2022) Functional characterization of bat IRF1 in IFN induction. Dev Comp Immunol 136:104500 35933044 10.1016/j.dci.2022.104500

[19] Banerjee A, Falzarano D, Rapin N, Lew J, Misra V (2019) Interferon regulatory factor 3-mediated signaling limits middle-east respiratory syndrome (MERS) coronavirus propagation in cells from an insectivorous bat. Viruses 11:152 30781790 10.3390/v11020152 PMC6410008

[20] Wang J, Lin Z, Liu Q, Fu F, Wang Z, Ma J, Wang H, Yan Y, Cheng Y, Sun J (2022) Bat employs a conserved MDA5 gene to trigger antiviral innate immune responses. Front Immunol 13:904481 35677039 10.3389/fimmu.2022.904481 PMC9168228

[21] Fu F, Shao Q, Zhang J, Wang J, Wang Z, Ma J, Yan Y, Sun J, Cheng Y (2023) Bat STING drives IFN-beta production in anti-RNA virus innate immune response. Front Microbiol 14:1232314 37744905 10.3389/fmicb.2023.1232314 PMC10514486

[22] Genin P, Lin R, Hiscott J, Civas A (2012) Recruitment of histone deacetylase 3 to the interferon-A gene promoters attenuates interferon expression. PLoS One 7:e38336 22685561 10.1371/journal.pone.0038336 PMC3369917

[23] Lin Z, Wang J, Zhao S, Li Y, Zhang Y, Wang Y, Yan Y, Cheng Y, Sun J (2022) Goose IRF7 is involved in antivirus innate immunity by mediating IFN activation. Dev Comp Immunol 133:104435 35562079 10.1016/j.dci.2022.104435

[24] Shi Z (2010) Bat and virus. Protein Cell 1:109–114 21203979 10.1007/s13238-010-0029-7 PMC4875169

[25] Lin HH, Horie M, Tomonaga K (2022) A comprehensive profiling of innate immune responses in Eptesicus bat cells. Microbiol Immunol 66:97–112 34842304 10.1111/1348-0421.12952

[26] Taniguchi T, Ogasawara K, Takaoka A, Tanaka N (2001) IRF family of transcription factors as regulators of host defense. Annu Rev Immunol 19:623–655 11244049 10.1146/annurev.immunol.19.1.623

[27] Antonczyk A, Krist B, Sajek M, Michalska A, Piaszyk-Borychowska A, Plens-Galaska M, Wesoly J, Bluyssen HAR (2019) Direct inhibition of IRF-dependent transcriptional regulatory mechanisms associated with disease. Front Immunol 10:1176 31178872 10.3389/fimmu.2019.01176 PMC6543449

[28] Sun BJ, Chang MX, Song Y, Yao WJ, Nie P (2007) Gene structure and transcription of IRF-1 and IRF-7 in the mandarin fish *Siniperca chuatsi*. Vet Immunol Immunopathol 116:26–36 17289159 10.1016/j.vetimm.2007.01.001

[29] Zhang YB, Hu CY, Zhang J, Huang GP, Wei LH, Zhang QY, Gui JF (2003) Molecular cloning and characterization of crucian carp (*Carassius auratus* L.) interferon regulatory factor 7. Fish Shellfish Immunol 15:453–466 14550671 10.1016/s1050-4648(03)00025-1

[30] Escalante CR, Yie J, Thanos D, Aggarwal AK (1998) Structure of IRF-1 with bound DNA reveals determinants of interferon regulation. Nature 391:103–106 9422515 10.1038/34224

[31] Kileng O, Bergan V, Workenhe ST, Robertsen B (2009) Structural and functional studies of an IRF-7-like gene from Atlantic salmon. Dev Comp Immunol 33:18–27 18778729 10.1016/j.dci.2008.07.020

[32] Al-Eitan L, Mihyar A (2024) The controversy of SARS-CoV-2 integration into the human genome. Rev Med Virol 34:e2511 38282406 10.1002/rmv.2511

[33] Al-Eitan L, Mihyar A, Zhang L, Bisht P, Jaenisch R (2024) Genomic and biological variation in bat IFNs: An antiviral treatment approach. Rev Med Virol 34:e2488 37921610 10.1002/rmv.2488

