# Supplementary material for: Conserved function of bat IRF7 in activating antiviral innate immunity: insights into the innate immune response in bats
Source: Vet Res. 2025 Mar 19;56:59. doi: 10.1186/s13567-025-01490-3 (PMC11921751; doi:10.1186/s13567-025-01490-3)
Supplement: Supplementary file 1 — Additional file 1. Sequences of Primers used in this study, including bat IRF7 cloning primers, bat IRF7 truncated plasmid primers, and quantitative RT‒PCR primers. [file 13567_2025_1490_MOESM1_ESM.docx]

**Additional file 1 Primers used in this study**

| **Genes** | **Primer** | **Nucleotide sequence of primers (5’-3’)** |
| --- | --- | --- |
| batIRF7 | Forward | ATGGCCTTGGACCCTGAGA |
|  | Reverse | TCACTGCTCCACCTCCAT |
| pcDNA3.1-batIRF7 | Forward | GTCCAGTGTGGTGGAATTcATGGCCTTGGACCCT |
|  | Reverse | CCTTGTAGTCCTCGAGCTGCTCCACCTCCATCA |
| batIRF7-dAA123-223 | Forward | GAGCTCTGCTCCAGTTACCTTCCGTGGGCTGTGGAGA |
|  | Reverse | ACTGGAGCAGAGCTCATACACTTTGTGCGG |
| batIRF7-dAA223-335 | Forward | CCTTCCGGGCAGCGGGTGCAGCCCTGCATCAAA |
|  | Reverse | CCGCTGCCCGGAAGGGAGCTCTGGACCAGT |
| batIRF7-dIB | Forward | CCCTATACCTGGCAGCAGGAGACGGTCGGG |
|  | Reverse | CTGCCAGGTATAGGGCTGCACCTCTGCTAGG |
| batIRF7-dIRF | Forward | TTGGACCCTGAGAGGGTGGGGTGCAGAGAAGATCCAG |
|  | Reverse | CCTCTCAGGGTCCAAGGCCATgAATTCCAC |
| batIRF7-dIRF-3 | Forward | GCAGAGCCCAGCCTGCGGGAAGGCGTGTCC |
|  | Reverse | CAGGCTGGGCTCTGCCTGCAGACCGAGCTG |
| batIRF7-dDUF | Forward | AGGAGCCTGGTCCTGCAGCTCGAGGACTAC |
|  | Reverse | CAGGACCAGGCTCCTCTCCTTGGGCCTCCC |
| qbatIFNβ | Forward | GCACCGGCTGGAATGAGACCA |
|  | Reverse | GTCCAGGCATTGGCTGT |
| qbatOAS1 | Forward | ATCTGCAGTTTCCTGAAGGAG |
|  | Reverse | GCTGAGGAAGCGACGAGGTC |
| qbatβ-actin | Forward | CCATCCTGCGTCTGGACCTGG |
|  | Reverse | GTGGCCATCTCCTGCTCGAAG |
| qbatIRF7 | Forward | TTCCGCTGCGCCCTGAGCAGC |
|  | Reverse | CTTCAAGGGTCTTGCTGT |
